# Role of Protein Glycosylation in Interactions of Medically Relevant Fungi with the Host

**DOI:** 10.3390/jof7100875

**Published:** 2021-10-18

**Authors:** Manuela Gómez-Gaviria, Ana P. Vargas-Macías, Laura C. García-Carnero, Iván Martínez-Duncker, Héctor M. Mora-Montes

**Affiliations:** 1 Departamento de Biología, División de Ciencias Naturales y Exactas, Campus Guanajuato, Universidad de Guanajuato, Noria Alta s/n, Guanajuato 36050, Gto., Mexico; manuela.gomezg8@gmail.com (M.G.-G.); ap.vargasmacias@ugto.mx (A.P.V.-M.); laura_cgc@hotmail.com (L.C.G.-C.); 2 Centro de Investigación en Dinámica Celular, Laboratorio de Glicobiología Humana y Diagnóstico Molecular, Instituto de Investigación en Ciencias Básicas y Aplicadas, Universidad Autónoma del Estado de Morelos, Cuernavaca 62209, Mor., Mexico; duncker@uaem.mx

**Keywords:** *N*-glycosylation, *O*-glycosylation, glycosylphosphatidylinositol anchors, host–fungus interaction, glycoproteins, virulence, pathogenicity, pathogenic fungi

## Abstract

Protein glycosylation is a highly conserved post-translational modification among organisms. It plays fundamental roles in many biological processes, ranging from protein trafficking and cell adhesion to host–pathogen interactions. According to the amino acid side chain atoms to which glycans are linked, protein glycosylation can be divided into two major categories: *N-*glycosylation and *O-*glycosylation. However, there are other types of modifications such as the addition of GPI to the *C-*terminal end of the protein. Besides the importance of glycoproteins in biological functions, they are a major component of the fungal cell wall and plasma membrane and contribute to pathogenicity, virulence, and recognition by the host immunity. Given that this structure is absent in host mammalian cells, it stands as an attractive target for developing selective compounds for the treatment of fungal infections. This review focuses on describing the relationship between protein glycosylation and the host–immune interaction in medically relevant fungal species.

## 1. Introduction

Protein glycosylation is a highly conserved post-translational modification found in both prokaryotic and eukaryotic cells characterized by the addition of oligosaccharides and glycolipids to the peptide backbone. Glycans influence biological processes, such as protein folding, targeted transport, cellular localization, and mediation of host–pathogen interactions, among others [1]. Glycosylation is carried out by different mechanisms that occur in different cell compartments. According to the amide or hydroxyl groups of amino acids to which glycans are attached, glycoproteins are divided into *N*-linked glycoproteins or *O*-linked glycoproteins, although other types of glycoproteins have been described that are not part of this work. In the former, glycans are linked to asparagine residues, contained within a consensus sequon, Asn-X-Ser/Thr (where X is any amino acid except for proline) [2]; meanwhile, in the latter, glycans are attached to a subset of serines, threonines, or hydroxylysines [3,4,5].

Another post-translational modification is the anchoring of cell membrane and cell wall proteins to glycosylphosphatidylinositol (GPI) covalently attached to the *C*-terminus. GPI biosynthesis is a multistep conserved pathway in eukaryotes that culminates in the generation of GPI glycolipids that anchor many proteins to the cell surface. GPI anchor synthesis appears to be essential in lower eukaryotes, affecting growth and viability [6]. Additionally, there is evidence that GPI-anchored proteins are important for the pathogenicity and virulence of many pathogens, as discussed in the following sections.

As mentioned, glycoproteins are involved in a wide range of biological processes which ensure cell stability [7]. For instance, they are a major constituent of the fungal cell wall and plasma membrane, and the biosynthetic pathways of these glycans have been thoroughly dissected in organisms such as *Candida albicans* and *Saccharomyces cerevisiae*. Rather than being a rigid and impenetrable shield, the fungal wall is plastic, permeable, and a molecular scaffold to display molecules to respond to changes in the environment or stresses. In addition, it is paramount for viability, morphogenesis, and pathogenesis [8]. This wall is composed of structural polysaccharides (glucans, chitin, and chitosan) and GPI-, *N*-linked, and *O*-linked glycoproteins [8]. Moreover, in fungal infections, the cell wall is one of the first points of contact with the host, being essential for fungal pathogenicity and virulence as well as the host’s immune response [9]. The cell wall plays a major role during fungal invasion. It is capable of inducing immune responses due to the components that are not found in the host cells, through the recognition of cell surface fungal-specific molecules, named pathogen-associated molecular patterns, by pattern recognition receptors [10]. Some of the wall antigens are also directly involved in fungal adhesion and colonization of the host tissues [11].

Since *N*-linked and *O*-linked glycoproteins, as well as GPI-anchored proteins (GPI-AP), are important secreted proteins, and cell wall and plasma membrane components, it is now well established that they contribute to fungal pathogenicity and virulence, and recognition by the host immunity. In this review, we provide an up-to-date view of these protein modifications in medically relevant fungal species.

## 2. The *Candida albicans* Protein Glycosylation Pathways

*C. albicans* is one of the most common human fungal pathogens that cause benign superficial infections of the mucosa and life-threatening systemic infections [12]. The deep-seated infection is associated with high mortality rates, and treatment is becoming challenging because of the increased number of isolates showing drug resistance to the conventional therapeutic options [12]. Therefore, the *C. albicans* cell wall biosynthetic routes, including protein glycosylation, and the interaction of the cell wall components with the host immunity have attracted attention in recent decades, with the idea to find targets for the development of new antifungal drugs or immunomodulatory therapies. Since this organism has been regarded as a biological model of a fungal pathogen, its protein glycosylation has been thoroughly studied, and here, we will take these pathways as an example to describe *N*-linked and *O*-linked glycosylation, and GPI anchor synthesis.

### 2.1. The N-Linked Glycosylation Pathway

**Figure 1:**
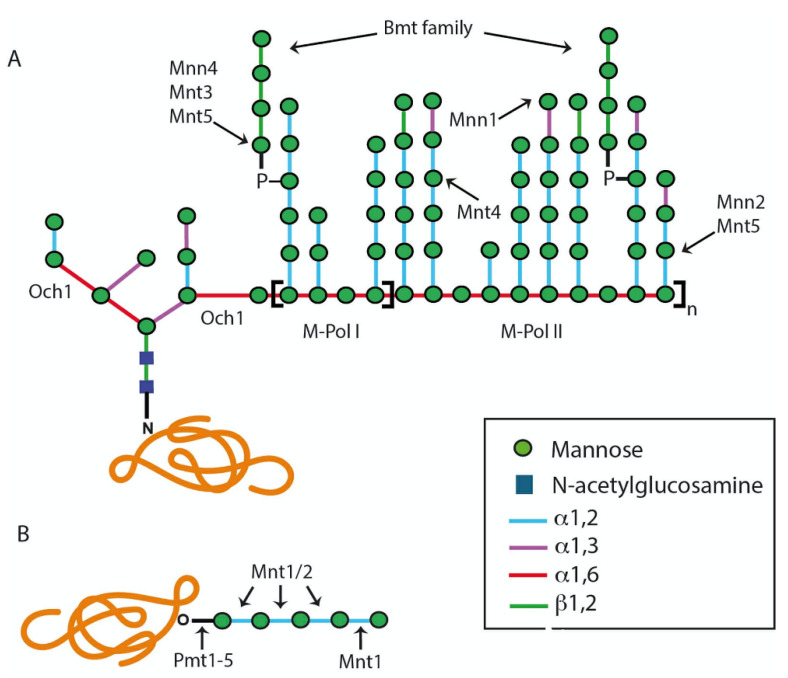
Schematic diagram representing the *Candida albicans N*-linked and *O*-linked glycans. (**A**) *N*-linked glycan. The outer chain elaboration begins with the addition of an α-1,6-mannose residue to the *N*-linked glycan core by the α-1,6-mannosyltransferase Och1. Then, an α-1,6-mannose backbone is synthesized by M-Pol I and M-Pol II complexes, and this is branched with α-1,2-mannooligomers that can be decorated with phosphomannan, β-1,2-mannose, or α-1,3-mannose units. (**B**) *O*-linked glycan. The addition of the first mannose residue is initiated in the endoplasmic reticulum by protein mannosyltransferases encoded by the *PMT* gene family. The glycoprotein is then transported to the Golgi complex for the addition of further mannose residues by mannosyltransferases Mnt1 and Mnt2.

The first steps of *N*-linked protein glycosylation are conserved among eukaryotes, such as fungi, plants, protozoa, and mammalian cells, although some peculiarities have been reported in particular species [13,14,15,16]. To obtain the final structure displayed on glycoproteins, *N*-linked glycans must undergo a series of modifications carried out by glycosidases and glycosyltransferases in the endoplasmic reticulum (ER) and the Golgi complex. This processing can be divided into two sequential stages: (a) the assembly of an oligosaccharide on an isoprenoid lipid in the rough ER by a set of specific glycosyltransferases, and its subsequent translocation to the nascent protein, and (b) further modification of an *N*-linked glycan by ER glycosidases and Golgi glycosyltransferases [13,17,18]. The enzymes involved in *N*-linked glycan synthesis are highlighted in Figure 1.

#### 2.1.1. Assembly of Lipid-Linked Oligosaccharide

The lipid moiety of the intermediates involved in protein glycosylation reactions is a polymer of isoprene units (CH3–C(CH3) = CH–CH2–), dolichol (Dol) [2,19,20]. In *C. albicans*, as in other eukaryotic cells, the synthesis of Dol-PP-GlcNAc_2_Man_5_ is achieved in the ER cytoplasmatic face by the addition of N-acetyl-D-glucosamine (GlcNAc) and mannose (Man), using UDP-GlcNAc and GDP-Man as donor substrates [21]. Each of the sugar residues is added to the lipid-linked precursor by a specific glycosyltransferase; Alg7 and Alg13 add phospho-GlcNAc to Dol phosphate, and Alg1, Alg2, Alg11, Alg3, and Alg12 add the first, second, third, fourth, and fifth phospho-Man residues, respectively [22,23]. The precursor is then flipped into the ER lumen in a process that involves the *RFT1* product [17]. Within the ER lumen, the mannosyltransferases Alg3, Alg9, and Alg12 add four more mannose residues to the lipid-linked precursor, using Dol-P-Man as the sugar donor, and then Alg6, Alg8, and Alg10 add three glucose (Glc) units to generate the final glycan precursor Dol-PP-GlcNAc_2_Man_9_Glc_3_ [2,13].

Once the precursor is assembled, it is transferred to the nascent protein by the oligosaccharyltransferase enzymatic complex [13,24]. *C. albicans* encodes all the subunit orthologs found in the *S. cerevisiae* oligosaccharyltransferase enzymatic complex, which is composed of nine different transmembrane subunits: Wbp1, Swp1, Stt3, Ost1, Ost2, Ost3, Ost4, Ost5, and Ost6, where Stt3 is the catalytic subunit [13,25].

#### 2.1.2. Modification of the *N*-Linked Glycan Core by Glycosidases and Transferases

Once the oligosaccharide has been transferred to the nascent protein, it is processed by ER α-glycosidases: α-glucosidases I and II, and α-1,2-mannosidase [18,26]. α-Glucosidases I and II remove the terminal α-1,2-linked and two remaining α-1,3-linked glucose residues, respectively, and mannosidase generates the isomer Man_8_GlcNAc_2_ [24]. In *C. albicans*, *CWH41* encodes α-glucosidase I, *ROT2* and *GTB1* encode the heterodimeric α-glucosidase II, and *MNS1* encodes the ER mannosidase [3,26].

Following the glycosylation steps in the ER, *N*-linked glycans are elongated by Golgi mannosyltransferases, which require GDP-Man as a sugar donor, and the transport of this compound into the lumen of the Golgi apparatus is mediated by a specific nucleotide sugar transporter encoded by *VRG4* [27]. The α-1,6-mannosyltransferase Och1 starts the addition of an α-1,6-mannose unit to the backbone of the *N*-linked glycan outer chain (Figure 1) [28]. Subsequently, the α1,6-mannose backbone is extended by the sequential action of the mannan polymerase I (Mnn9 and Van1) and mannan polymerase II (Mnn9, Anp1, Mnn10, Mnn11, and Hoc1) enzyme complexes [13,24,28]. This α-1,6-mannose backbone is branched with α-1,2-oligomannans by the action of mannosyltransferases Mnt3, Mnt4, Mnt5, Mnn2, and Mnn5, and this may be capped with terminal α-1,3-mannoses by the action of the mannosyltransferase Mnn1 (Figure 1) [13]. In *C. albicans*, it is frequently found that the α-1,2-mannose branches are further β-mannosylated, a mechanism that is carried out by the *BMT* gene family [29]. Additionally, in this organism, the *N-*linked glycans can be modified by mannosyl-phosphate moieties, named phosphomannans, which can work as molecular scaffolds to synthesize α-1,2-mannoligosaccharides [24], in a process that involves the *MNN4*-like gene family and the mannosyltransferases Mnt3 and Mnt5 [30,31].

### 2.2. The O-Linked Glycosylation Pathway

In *C. albicans*, the *O*-linked glycans are linear oligosaccharides of one to seven α-1,2-linked mannose residues [32]. The addition of α-linked mannose residues to the serine or threonine residues is initiated in the ER lumen, using Dol-P-Man as a sugar donor, in a reaction catalyzed by any of the five members of the protein mannosyltransferase (*PMT)* gene family [13,33]. Then, glycoproteins are transported to the Golgi complex to start the addition of additional mannose residues by the action of Golgi α-1,2-mannosyltransferases encoded by *MNT1* and *MNT2* [32,34]. These are GDP-mannose-dependent mannosyltransferases that may have redundant activities but with a preference to add particular mannose residues to the *O*-linked glycans: Mnt1 adds the second mannose, whereas Mnt2 adds the third mannose unit during the elongation step (Figure 1) [32]. These *O*-linked glycans can also be phosphomannosylated by Mnt3 and Mnt5 [31].

### 2.3. Glycosylphosphatidylinositol Anchor Synthesis

**Figure 2:**
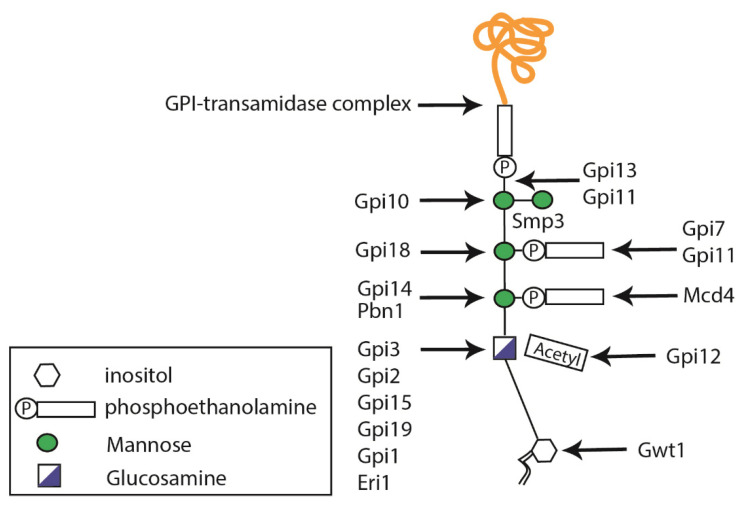
Schematic diagram representing glycosylphosphatidylinositol (GPI) anchor synthesis. GPIs are composed of an inositol molecule that is modified in the endoplasmic reticulum. The Smp3 protein is responsible for adding the fourth mannose residue, which is important for protein transfer.

GPI anchors are structurally complex glycophospholipids that are post-translationally attached to the *C*-terminal end of proteins containing the appropriate GPI signal sequence [35]. The first step in GPI biosynthesis takes place in the ER, where a phosphatidylinositol receives GlcNAc from UDP–GlcNAc, in a reaction catalyzed by a multi-subunit GPI-N-acetylglucosaminyltransferase (GPI-GnT) complex [6], whose catalytic subunit is encoded by *GPI19* (Figure 2) [13,36]. Then, the inositol-acyl group is removed during GPI maturation, triple mannosylated, and decorated with two–three phosphoethanolamine groups at the different mannoses (Figure 2) [6]. In fungi, the addition of a fourth mannose residue is required and catalyzed by the mannosyltransferase Smp3 [37,38]. The complete GPI-anchored protein is formed once the anchor is attached to a new translocated protein in the ER lumen, via the GPI transamidase complex composed of Gaa1, Gpi8, and Gpi16 [39], and is then transported via the secretory pathway to the cell wall or plasma membrane.

## 3. Protein Glycosylation in Other Medically Relevant Fungal Pathogens

In general terms, the dissected models of *C. albicans* protein glycosylation can be applied to other fungal species, although species-specific glycans have been isolated from clinically relevant yeasts and molds.

In *A. fumigatus*, the cell wall contains galactomannan, which is a linear polysaccharide of α-1,2- and α-1,6-mannose units and branched with β-1,5- and β-1,6-galactofuranose residues [40], and is covalently linked to *N*-linked and *O*-linked glycans, β-(1,3)-glucans, and cell membrane GPI anchors [40,41]. Galactofuranose-containing glycans are peculiar and not synthesized by the human host. The first step in their synthesis involves the generation of UDP-galactofuranose, by the action of a cytosolic UDP-galactopyranose mutase, encoded by *ugm1* [42]; then, a UDP-galactofuranose transporter, GlfB, imports this activated sugar into the Golgi lumen, where the galactofuranosyltransferase GfsA binds this sugar moiety to glycans [43,44,45]. The mannose-rich core of galactomannan is also synthesized in the Golgi complex, and the α-1,2-mannosyltransferases ktr4 and ktr7 have been implicated in this biosynthetic process [46,47]. Since a *glfB*Δ mutant did not show galactosylated high-mannose *N*-linked glycans, it was proposed that this transport is required for galactofuranosylation of *A. fumigatus N*-linked glycans [45]. Even though it is not covalently linked to proteins by the canonical protein glycosylation pathways, it is worthy of mention that galactosaminogalactan, a galactopyranose-containing glycan, is in the surface of *A. fumigatus* mycelia [48]. In addition to galactopyranose, it also contains galactosamine and N-acetylgalactosamine bound via α-1,4-linkages [48].

The generation of an *A. fumigatus* undecuple mutant, lacking *och1-1*, *och1-2*, *och1-3*, *och1-4*, *mnn9*, *van1*, *anp1*, *mnn10*, *mnn11*, *mnn2*, and *mnn5*, putative orthologs of genes encoding for α-1,2- and α-1,6-mannosyltransferases, did not show a reduction in the hyphal wall mannan content, but this was affected when the conidial cell wall was analyzed [49], suggesting that canonical genes, previously assigned to the protein glycosylation pathways, may have different functions in molds, and that their function is tightly regulated by cell morphology and likely by environmental cues.

Different *N*-linked and *O*-linked glycans have also been reported in *Cryptococcus* spp. There is strong evidence suggesting that the *N*-linked glycan core is similarly synthesized in both *C. albicans* and *C. neoformans*, but the enzymes involved in the synthesis of the *N*-linked outer chain seem to be absent from the *C. neoformans* genome [50], suggesting no high-mannose *N*-linked glycans are synthesized in this organism. However, they may contain sialic acid or xylose [51,52]. Different types of *O*-linked glycans have been characterized in *Cryptococcus laurentii*: linear α-1,2-mannotriose and oligomannose chains modified with xylose, and extended chains of α-1,6-galactose linked to a single mannose residue [53]. In the case of *C. neoformans*, the most abundant *O*-linked glycans are α-1,2-mannans but connected by an α-1,6-mannose at the third position of the glycan [54]. As a minority, xylose-modified *O*-linked glycans are also present [54]. Analysis of the biosynthetic machinery behind these structures indicated that the α-1,2-mannosyltransferase Ktr3 is in charge of adding the second mannose residue, and the α-1,6-mannosyltransferases Hoc1 and Hoc3 add the third mannose unit to xylosylated and non-xylosylated *O*-linked glycans, respectively [54]. Finally, xylose is transferred to glycans from UDP-xylose, which is synthesized by the *UXS1* product [54], and *XPT1* that codes for a Golgi-resident xylosyltransferase [55].

In *Sporothrix schenckii*, rhamnose-containing *N*- and *O*-linked glycans have been reported and contain terminal α-1,2-, α-1,3-, or α-1,4-rhamnose units [56]. In addition, *O*-linked glycans mainly contain glucuronic acid, which may be mono- or bi-rhamnosylated [56]. Protein rhamnosylation depends on the presence of *RmlD*, a gene encoding for an epimerase/reductase essential for the elaboration of UDP-rhamnose, the sugar donor in this post-translational modification [57]. Moreover, sialic acid- and galactose-containing *N*-linked glycans have been reported in the *S. schenckii* cell wall [58].

## 4. Relevance of Fungal Protein Glycosylations during Host–Pathogen Interaction

In pathogenic fungi, many proteins are involved in the *N*-linked and *O*-linked glycosylation processes as well in GPI anchor synthesis. Both are important protein modifications in fungi such as *C. albicans*, *A. fumigatus*, and *C. neoformans*, among others, because they intervene in pathogen–host interactions and can directly or indirectly affect virulence [59,60].

The most used strategy to assess the contribution of particular genes to both protein glycosylation and the interaction with the host is the generation of mutant strains and the comparison of their phenotype with the parental strains. In this section, we provide a summary of the contribution of the protein glycosylation pathways to host–fungus interactions (Table 1).

### 4.1. Relevance of N-Linked Glycosylations during Host–Pathogen Interaction

In *C. albicans*, the ER α-glucosidase I, α-glucosidase II, and α1,2-mannosidase, encoded by *CWH41*, *ROT2,* and *MNS1*, respectively, are in charge of the *N*-linked glycan core processing in the ER before transport to the Golgi complex [26]. The *C. albicans cwh41*Δ and *rot2*Δ showed cellular aggregates and defects in the cell wall composition, changes that led to virulence attenuation in a murine model of systemic candidiasis, and to a reduced ability to stimulate cytokine production by human peripheral blood mononuclear cells (PBMCs) (Table 1) [26]. In the case of the *C. albicans mns1*Δ null mutant, fewer phenotypical changes were observed when compared with the mutants with defects in the ER glucosidases, but virulence and stimulated cytokine profiles were still affected (Table 1) [26,63]. These results underscore the relevance of proper *N*-linked glycan processing for the *C. albicans*–host interaction. In *A. fumigatus*, Af*cwh41* deletion generated defective processing of *N*-linked glycans, a reduction in conidia formation, and changes in the cell wall composition, polar growth, and hyphal elongation (Table 1) [61]. However, this gene was not required for virulence [61], contrary to *C. albicans*. Currently, there are no reports about the function of the homologs of *C. albicans ROT2* and *MNS1* in *A. fumigatus* and *C. neoformans*.

*OCH1* disruption, which encodes the α-1,6-mannosyltransferase that begins the synthesis of the *N*-linked glycan outer chain, has demonstrated to be a valuable tool to understand the importance of fully extended *N*-linked glycans during the interaction with the host [28,64,65]. The *C. albicans och1*Δ null mutant showed defects in the cell wall and a decreased growth rate and tended to form aggregates [28]. Importantly, the absence of this gene caused virulence attenuation in a murine model of systemic infection, even though the fungus was able to colonize some organs [28]. Moreover, these mutant cells showed a reduced ability to stimulate cytokine production by human PBMCs, dendritic cells, and macrophages (Table 1) [63,100,101,102]. In *Candida parapsilosis*, another opportunistic pathogen, loss of *OCH1* caused large changes in the cell morphology, cell wall composition, and susceptibility to cell wall perturbing agents (Table 1) [65]. As with *C. albicans*, the *C. parapsilosis och1*Δ null mutant showed a reduced ability to stimulate cytokine production by human PBMCs, and virulence attenuation in neonate and adult murine models of systemic infections, but not in the *Galleria mellonella* invertebrate model (Table 1) [65,66]. In *S. schenckii, OCH1* silencing caused minimal changes in the cell phenotype, with only subtle changes in the cell wall composition (Table 1) [64]. However, it was reported that as a compensatory mechanism for the loss of the *N*-linked glycan outer chain, these mutants increased the cell wall *O*-linked glycan content [64,103]. The contrasting phenotype between these mutant strains and those generated in *C. albicans* is likely because glycoproteins that have a high mannose content may not be the most abundant element in the *S. schenckii* cell wall [64]. Nevertheless, silenced strains showed virulence attenuation in a murine model of systemic infection and the invertebrate models *G. mellonella* and *Tenebrio molitor* [64,67] and had a diminished ability to stimulate proinflammatory cytokines when interacting with PBMCs, but an increased ability to stimulate IL-10 (Table 1) [64]. Contrasting these observations, the *A. fumigatus och1*Δ mutant did not show significant changes in growth or the cell wall composition, but a defect in its ability to sporulate (Table 1) [68]. Virulence analysis in a murine model did not show virulence attenuation, suggesting that fully extended *N*-linked glycans are not associated with *A. fumigatus* virulence [68,69]. In *C. neoformans, OCH1* disruption slightly attenuated virulence when compared to the control strain (Table 1) [70]. These data suggest that, despite the fact the gene has a conserved role in protein glycosylation, it has a species-specific impact on the host–fungus interaction, suggesting that fully extended *N*-linked glycans may play different roles on the surfaces of medically relevant fungal species.

Many mannosyltransferases participate in the *N*-linked glycosylation process, and members of the MNN families fulfill different functions within the pathway [13,18]. The *C. albicans MNN1* family encodes α-1,3-mannosyltransferases that add terminal mannose residues to the branches of the *N*-linked glycan outer chain and is composed of six members, *MNN1*, *MNN12*, *MNN13*, *MNN14*, *MNN15*, and *MNN16* [71]. Null mutants of the members of the *MNN1* family have been generated, and it was observed that all mutants had similar growth rates compared to the wild-type strain, with no obvious changes in the morphology and cell wall composition, suggesting functional redundancy [71]. The only exception was *MNN14*, where the null mutant showed a subtle defect in the cell wall composition, indicating that it is the dominant member of the gene family (Table 1) [71]. Despite the fact the *mnn14*Δ mutant phenotype was not as severe as the one reported for *OCH1*, *ROT2*, or *CHW41* disruption, the mutant showed virulence attenuation in a murine model of disseminated candidiasis but did not show defects in cytokine production [71], suggesting that the structural changes in the *N*-linked glycans were not sufficient to disrupt the ligand–receptor interactions between the fungal and immune cell surfaces but affected the cellular fitness to display full virulence. As previously mentioned, the *MNN2* gene family encodes α-1,2-mannosyltransferases involved in the synthesis of the backbone lateral chains of the *N*-linked glycan outer chain and is composed of *MNN2*, *MNN21*, *MNN22*, *MNN23*, *MNN24*, and *MNN26* [72]. Mutants of this family showed changes in phenotype, and it was established that Mnn2 and Mnn26 are necessary for the addition of the initial α-1,2-mannose residue to the α-1,6-mannose backbone, and Mnn21, Mnn22, Mnn23, and Mnn24 are required to add α-1,2-mannose units to the α-1,6-α-1,2-mannose scaffold [72]. The absence of these genes caused severe virulence attenuation in both *G. mellonella* and murine models of systemic infection, suggesting the lateral chains of the *N*-linked glycan outer chain backbone are relevant for the pathogen–host interaction (Table 1) [72]. Similar to *C. albicans*, in *C. neoformans*, *MNN2* encodes for a mannosyltransferase that adds mannose units to the *N*-linked glycan outer chain [70]. However, its contribution to the *C. neoformans* interaction with the host has not been elucidated yet.

In *C. albicans*, there is an extensive family of *MNN4*-like genes that is composed of *MNN41*, *MNN42*, *MNN43*, *MNN44*, *MNN45*, *MNN46*, and *MNN47*, which are presumed to be required for phosphomannan production [30]. Disruption of this gene family did not show any phenotype different from that described for *MNN4*, suggesting this gene is the dominant member of the gene family [30]. Loss of Mnn4 did not affect virulence in a murine model of systemic candidiasis, but the null mutant showed virulence attenuation in *G. mellonella* larvae, similar to the *Candida tropicalis mnn4*Δ null mutant, suggesting that phosphomannosylation is required to adapt to this host milieu [73,74]. Furthermore, loss of *MNN4* led to changes in the *C. albicans*–macrophage interaction, where fungal uptake was reduced, and to increased resistance to antimicrobial peptides, stressing the relevance of this glycan moiety in the *C. albicans*–immune effector interaction (Table 1) [30,73,104,105].

Another important mannosyltransferase that participates in protein glycosylation is the α-1,2-mannosyltransferase Mnn5, which is regulated by iron in *C. albicans* [75]. An *mnn5*Δ null mutant showed hypersensitivity to wall perturbing agents, inability to undergo dimorphism, and virulence attenuation in a murine model of systemic candidiasis (Table 1) [75].

After the addition of the first α-1,6-mannose residue to the backbone of the *N*-linked glycan outer chain by Och1, Mnn9 is thought to be the major contributor to the α-1,6 backbone extension [76]. Mutations in its encoding gene led to changes in the fungal cell wall, osmotic instability, slow growth rates, defective hyphae, and severe cell agglutination (Table 1) [76]. Despite the fact the virulence of this mutant strain has not been evaluated, it is suggested that it may show virulence attenuation, as with cells lacking Och1 or other relevant mannosyltransferases involved in *N*-linked glycan elaboration. In *A fumigatus*, Mnn9 is also a GDP-Man-dependent α-1,6-mannosyltransferase, and disruption of the encoding gene led to cell wall integrity defects, changes in morphogenesis, and sensitivity to some wall perturbing agents (Table 1) [77]; however, its relevance in the *Aspergillus*–host interaction remains to be investigated. Similar to Mnn9, Mnn10 is an α-1,6-mannosyltransferase responsible for the elongation of the *N*-linked glycan outer chain backbone. In *C. albicans*, *MNN10* deletion resulted in an abnormal organization of the cell wall, with a lower mannose content and increased exposure of β-1,3-glucan on the cell surface [78]. *MNN10* was shown to be required for *C. albicans* pathogenicity in a murine model of systemic infection; however, the null mutant did not show defects in the ability to invade the host tissues (Table 1) [78]. Instead, the diminished virulence was attributed to greater immunological recognition of the mutant strain by the host [78]. *MNN11* encodes for an α-1,6-mannosyltransferase involved in the final stage of *N*-linked glycoprotein elaboration [13]. Interestingly, disruption of this gene in *Candida glabrata* did not affect virulence (Table 1) [79].

Another important family for both *N*-linked and *O*-linked protein glycosylation is the *KRE2*/*MNT1* family, which encodes a set of five Golgi-resident type-II α-mannosyltransferases in *C. albicans* [31,32,34]. Mnt3, Mnt4, and Mnt5 play a role in *N*-linked glycosylation [31], while Mnt1 and Mnt2 play a role in the synthesis of *O*-linked glycans [34]; the latter will be addressed in the following section. A triple mutant, *mnt3*-*mnt4*-*mnt5*Δ, exhibited an aggregated phenotype, increased susceptibility to cell wall perturbing agents, a significant reduction in the ability to stimulate cytokine production by PBMCs, and virulence attenuation in a murine model of systemic candidiasis (Table 1) [31]. These data indicate that this gene family is as relevant for interaction with the host as the *MNN* gene families involved in *N*-linked glycan synthesis.

In *C. albicans*, the *BMT* gene family is composed of nine members, *BMT1-9*, and encodes for β-mannosyltransferases responsible for adding β-mannose to the phosphopeptidomannan (PPM) [29]. The *bmt*Δ null mutants did not show any defects in phenotype or growth, nor were they sensitive to chemical or antifungal compounds. Despite the fact that all strains showed an altered pattern of β-mannose epitopes in PPM [29], more studies are needed to determine the relevance of these genes in *C. albicans* pathogenicity.

Another gene that is involved in both *N*-linked and *O*-linked glycosylation is *PMR1*. In *C. albicans*, it is known that this gene acts as a Golgi-resident P-type ATPase ion pump and is responsible for providing the Mn^2+^ cofactor to mannosyltransferases [80]. The *C. albicans pmr1*Δ null mutant showed cell aggregation, defects in the cell wall composition, hypersensitivity to wall perturbing agents, constitutive activation of the cell wall integrity pathway, and attenuated virulence in a murine model of infection, all mice survived throughout the experiment, and colonization in the organs by the mutant was not observed (Table 1) [80]. This indicates that *PMR1* is essential for *C. albicans* virulence. In *Candida guilliermondii,* a low-virulence species, *PMR1* disruption affected cell growth and morphology, the cell wall, biofilm formation, and the interaction with PBMCs and, as expected, led to virulence attenuation in both *G. mellonella* and mice (Table 1) [81]. In *A. fumigatus*, *pmrA* disruption generated defects in growth, and changes in the wall composition, presenting an increased content of β-glucan and chitin [82]. Additionally, the null mutant was hypersensitive to cell wall inhibitors, but its virulence was not attenuated in a murine model of invasive aspergillosis [82], suggesting that PmrA is necessary for the *A. fumigatus* cell wall integrity but not for virulence and pathogenesis (Table 1). Other genes that are related to the glycosylation processes of *C. albicans* are *RER2*, *SRT1,* and *CWH8*. The Rer2 and Srt1 proteins represent cis prenyltransferases, which are responsible for carrying out the synthesis of the dolichol skeleton [106]. The *RER2* absence hinders the synthesis of dolichol in *C. albicans* and causes defects in growth, dimorphism, and the formation of the cell wall. In addition, *RER2* mutants are sensitive to antifungal agents [106]. Finally, *CWH8* encodes a dolichol pyrophosphate phosphatase that acts as a carbohydrate transporter during *N*-linked glycosylation and is responsible for converting dolichol pyrophosphate to dolichol phosphate in fungi [107]. Gene deletion disrupted this recycling pathway and pleiotropically affected the cell phenotype [107].

Collectively, all these studies show that many of the genes involved in the *N*-linked protein glycosylation pathway are important for host interaction and virulence.

### 4.2. Relevance of O-Linked Glycosylation during Host–Pathogen Interaction

*O*-Linked glycosylation has been studied extensively in *C. albicans*. This organism possesses a gene family known as *PMT*, which encodes five protein mannosyltransferases, Pmt1, Pmt2, Pmt4, Pmt5, and Pmt6, which initiate this post-translational modification [33,84]. The importance of this gene family for virulence and morphology has been evaluated by generating null mutant strains. All the single null mutants, except for *pmt5*Δ, showed defects in morphogenesis, likely attributed to the lack of mannosylation and, consequently, to a loss of activity of the target Pmt proteins, which are necessary for the polarized growth (Table 1) [84]. These mutants also showed remarkable susceptibility to antifungal agents; in particular, *pmt1*Δ and *pmt4*Δ were hypersensitive to antifungal agents, indicating that both proteins are involved in processes and structures that lead to high resistance to antifungal drugs (Table 1) [84]. The mutants also showed hypersensitivity to cell wall perturbing agents and changes in the cell wall components [84], and virulence was severely affected in the *pmt1*Δ, *pmt4*Δ, and *pmt6*Δ mutants when used to challenge a mouse model of systemic infection (Table 1) [85,86]. Pmt2 is essential for growth, and the *pmt5*Δ mutant did not exhibit decreased virulence in a systemic candidiasis model, but in other models of localized and systemic infection, it was demonstrated that *PMT5* has an incomplete but significant effect on *C. albicans* virulence [84,89]. Mutants in *PMT1*, *PMT4,* and *PMT2* are partially defective in protease secretion, which could limit the fungal ability to use host proteins as a nitrogen source, thus affecting fungal growth and fitness [60]. In *A. fumigatus*, the *PMT* gene family comprises only three genes, Af*pmt1*, Af*pmt2,* and Af*pmt4* [87,108]. Loss of Af*pmt1* resulted in growth defects, changes in the cell wall, and defects in cell morphology and conidia formation (Table 1) [108]. Interestingly, despite a reduction of about 60% in the *O*-linked glycan content, the Af*pmt1*Δ mutant showed no defects in virulence when analyzed in a mouse model of infection [108], and a similar observation has been reported for Af*pmt4* (Table 1) [87]. In *C. neoformans*, the *PMT* family is also composed of three members, *PMT1*, *PMT2*, and *PMT4* [88]. Disruption of *PMT4* generated dramatic effects on fungal virulence, morphological defects, and alterations in the cell wall [88,109]. *PMT1* disruption led to virulence attenuation in a macrophage death model and a murine model of cryptococcosis by inhalation (Table 1) [88].

As mentioned previously, another gene family important for *O*-linked glycosylation is *KRE2*/*MNT1*. From this, only *MNT1* and *MNT2* participate in this biosynthetic pathway in *C. albicans* [34]. The *mnt1*Δ null mutant showed changes in cell morphology, an inability to adhere to human buccal epithelial cells, hypersensitivity to wall perturbing agents, and virulence attenuation in a murine model of systemic infection (Table 1) [34,90]. The *mnt2*Δ null mutant exhibited a less marked phenotype than the *mnt1*Δ mutant but showed virulence attenuation (Table 1) [34]. In *A. fumigatus*, loss of Af*mnt1* reduced the cell wall thickness and decreased cell growth, the ability to form conidia, and virulence in a murine infection model (Table 1) [110]. *C. neoformans* appears to have only one *MNT1* ortholog, but this has not been characterized [50].

Thus, it can be concluded that *O*-linked glycosylation is an important event for cell fitness and for displaying full virulence.

## 5. Relevance of Glycosylphosphatidylinositol Anchors during Host–Pathogen Interaction

GPI biosynthesis has been reported to be important not only for the integrity of the cell wall but also for the fungal ability to cause damage to the host [6,111]. To date, only a few genes that participate in this pathway have been reported to be important for the host–fungus interaction. *GWT1* and *MCD4* have been described as part of the *C. albicans* GPI synthesis. *GWT1* codes for an inositol acyltransferase that catalyzes the inositol acylation of glucosamine phosphatidylinositol (GlcN-PI) in the first step on the ER luminal side, having an important function in maintaining the cell wall integrity and enabling fungal adhesion to the host [6,91,92]. *MCD4* codes for a mannose-ethanolamine phosphotransferase that adds a phosphoethanolamine on the first mannose of the GPI core [6,92]. When the activity of these proteins is disrupted by GPI inhibitors in *Candida* spp., *Aspergillus* spp., *Fusarium* spp., and *Scedosporium* spp., a defective phenotype in the assembly, translocation, and maturation of GPI proteins is observed, affecting the cell wall integrity and structure, growth, dimorphism, and virulence (Table 1) [93,94]. For example, when *C. albicans* is treated with BIQ, a Gwt1 inhibitor, the mannoprotein expression in the cell surface is blocked, therefore inhibiting fungal adhesion to a monolayer of mammalian epithelial cells [112]. Gene overexpression overcame the defective phenotype, and downregulation of expression caused an increase in the sensibility to the inhibitor, obtaining a phenotype with reduced growth and morphological changes [112]. Additionally, when *C. albicans* is treated with E1210, another Gwt1 inhibitor, a reduced expression of GPI-anchored adhesins that are important virulence factors, such as members of the Als family, is observed, with additional defects in germ tube and biofilm formation [92]. When gepinacin is used, a similar effect is observed, since the target for this inhibitor is the same, causing a phenotype with reduced adherence, growth, dimorphism, and secretion of lytic enzymes (Table 1) [6]. Gepinacin causes this effect by stressing the ER due to a blockage of the GPI-anchored trafficking [6]. Several inhibitors have also been reported for Mcd4, including M743 and M720, that, although being highly efficient as antifungals, also inhibit GPI biosynthesis in mammalian cells [113].

From all of the known *S. cerevisiae* genes that participate in GPI synthesis, only one has been proven to be non-essential, *GPI7*, whose deletion caused cell wall and mating defects [114]. This gene was later studied in *C. albicans*, and it was found that deletion mutants showed defects in chlamydospore formation and dimorphism on solid media but normal growth in liquid [95]. The cell wall, cytokinesis, and cell shape were affected in both solid and liquid media, and in vivo assays showed a reduced virulence due to an inability to colonize the gastrointestinal tract in a mouse model of disseminated candidiasis [95]. Additionally, a higher sensibility to the macrophages’ lytic action was observed, probably due to a downregulation of Erk1/2 phosphorylation after phagocytosis [95]. The addition of ethanolamine phosphate on the second mannose of the GPI anchor was later proved to be essential for the transport and localization of the cell wall GPI proteins in *A. fumigatus* [6]. As in *C. albicans*, deletion of *gpi7* caused abnormal polarity and growth, altered levels of plasma membrane GPI proteins, incorrect localization of GPI proteins, and autophagy, but unlike *C. albicans*, no defects in the cell wall nor accumulation of GPI proteins in the ER were observed, and only the production of enlarged vacuoles and autophagosomes were present in the mutants, which are mechanisms to avoid ER stress and cell death (Table 1) [115].

*C. albicans GPI2* and *GPI19*, accessory subunits from the GPI-GnT complex, participate in morphogenesis and fungal cell wall biogenesis [116]. These two proteins have oppositive effects and negatively regulate each other: Gpi2 determines hyphal morphogenesis through Ras1 signaling, while Gpi19 controls ergosterol biosynthesis by downregulating *ERG11* levels and modulates the azole response [117,118]. A third subunit has been described, *GPI15*, a master activator of *GPI2* and *GPI19*, important for growth, cell wall integrity, and GPI synthesis in *C. albicans*. *GPI15* mutants were hypofilamentous, ergosterol defective, and azole sensitive and exhibited the phenotypes of both *GPI2* and *GPI19* mutants [96]. Additionally, *GPI15* mutants were less virulent in vitro, since this gene regulates hyphal filamentation and invasive growth, which are important for the establishment of the infection by *C. albicans* (Table 1) [96]. Thus, by controlling Gpi12 and Gpi19 activities, Gpi15 may be a good antifungal target.

Unlike what has been seen in *S. cerevisiae*, in *A. fumigatus*, the loss of GPI anchors is not lethal. PigA (Gpi3), the catalytic subunit of the GPI-GnT complex, is necessary for cell wall synthesis and virulence, as demonstrated by the generation of null mutants [97]. These mutants were capable of growing at a temperature from 30 to 50 °C, being the first report of a GPI deficiency that is not temperature sensitive, although their growth rate was significantly slower [97]. The mutant cells were more sensitive to SDS, which suggested an unstable or damaged cell membrane, and had increased levels of the chitinase ChiB, which is responsible for the degradation of chitin during autolysis (Table 1) [97]. Finally, reduced virulence was observed in a mouse model of invasive aspergillosis, probably caused by the lack of important virulence factors such as Ecm33 and Gel2 [97,119].

GPI synthesis has also been implicated, although indirectly, in *C. neoformans* virulence, as demonstrated by gene expression analyses. When *C. neoformans* was grown under iron limitation conditions, elevated expression was observed for several genes, including *CGP60*, a gene essential for capsule production, *FTR1*, an iron permease, and *GPI8* that encodes a GPI-transamidase [120]. When *C. neoformans GPI8* disruption was attempted, no mutant alleles were obtained in 192 transformants, suggesting that *GPI8* is essential for this organism [120]. This could be explained by the fact that the capsule is bound to cell wall components that can be affected by the lack of *GPI8*, suggesting this protein is important during cryptococcal infection [120].

## 6. Relevance of Non-Conventional Glycosylation Processes during Host–Pathogen Interaction

In the *A. fumigatus glfB*Δ mutant, the absence of galactofuranose showed a clear link between galactomannan and interaction with the host. The phenotype of the *glfB*Δ mutant was similar to the *umg1*Δ mutant, where poor growth was observed at the mammalian body temperature, showing attenuated virulence in a mouse model of invasive aspergillosis (Table 1) [45,98]. The *umg1*Δ mutants also showed a thinner cell wall that could be related to greater susceptibility to antifungal agents [98]. Disruption of *A. fumigatus ugm1* led to the elimination of galactofuranose synthesis, and cells became hyper-adherent to inherent surfaces and also to pulmonary epithelial cells [99]. This was explained by the fact that galactofuranose residues are capable of masking some polar groups during cell wall maturation, and therefore adherence can be moderated [99,121]. The contribution of this hyper-adherence to the virulence of the fungus is not clear, but it has been found that *ugm1*Δ mutants do not present changes in virulence in a murine model [99]; meanwhile, another study showed that these mutant cells had attenuated virulence in a murine model of invasive aspergillosis [98]. More studies are needed to determine whether Ugm1 is involved in *A. fumigatus* virulence. It is believed that galactomannan and galactofuranose do not fulfill the role of adhesins, but both can modulate the expression and exposure of other types of molecules that are responsible for mediating adherence in the fungus [121]. Loss of *ktr4* and *ktr7* led to an absence of galactomannan in the *A. fumigatus* cell wall, leading to severe phenotype defects, showing a hyperbranched mycelium, loss of polarity during conidia germination, and hyphal elongation [46]. In a murine model of invasive aspergillosis, it was determined that the absence of Ktr4 and Ktr7 severely affected virulence (Table 1) [46].

In *C. neoformans*, *KTR3* disruption, the mannosyltransferase involved in *O*-linked glycan synthesis [54], increased the sensitivity to SDS and NaCl, and virulence was attenuated in a murine model of cryptococcosis [54]. This virulence defect could be associated with changes in the function of cell surface and secretory proteins, particularly those that are associated with host infection, as a result of *O*-linked glycan involvement (Table 1) [54].

Silencing of *S. schenckii RmlD*, involved in protein rhamnosylation, did not affect cell growth or morphology but affected the cell wall composition and organization [57]. The silenced mutant strains exhibited a different ability to stimulate cytokine production by human PBMCs, stimulating lower levels of TNFα and IL-6, but a higher production of IL-1β and IL-10, compared to the wild-type strain [57]. Moreover, the silenced mutants showed virulence attenuation in the *G. mellonella* infection model, indicating that rhamnose is required to kill the larvae (Table 1) [57].

## 7. Protein Glycosylation as Potential Targets of Antifungal Drugs

As mentioned previously, the fungal cell wall is attractive for finding new targets for antifungal drugs because the molecular nature of its compounds is different from the proteins, polysaccharides, and oligosaccharides found in the mammalian host, and because some of these are ubiquitous molecules found in fungal cell walls, such as glucans and chitin, providing the alternative to have wide-ranging targets. Because of this, the synthesis of β-1,3-glucan and chitin has been thoroughly studied, and we currently have antifungal drugs that target these metabolic pathways available in the market, or in experimental phases [122]. However, since the synthesis of *N*-linked and *O*-linked glycans and GPI involves enzymes that are not present in human cells, such as those participating in glycan elongation in the Golgi complex, and due to the fact that it also contains bypassing steps not found in fungal cells [13] and that the human and pathogen glycosidases have different sensitivities to inhibitors [123], these biosynthetic pathways have also been analyzed as potential antifungal targets.

Rhodanine-derived benzylidene thiazolidinediones have shown mannosyltransferase inhibition that prevents the synthesis of fully extended *O*-linked mannans in *C. albicans*, and as a consequence, cells lost wall integrity and viability [122]. The derivative compound 5-[[3-(1-phenylethoxy)-4-(2-phenylethoxy)phenyl]methylene]-4-oxo-2-thioxo-3-thiazolidineacetic acid showed an in vitro ability to inhibit *C. albicans* Pmt1 and fungal proliferation, with an IC_50_ of 0.2–0.5 µM [124]. Despite the fact that human cells contain POMT1 and POMT2, enzymes with Dol-P-Man:protein O-D-mannosyltransferase activity, similar to that described for *C. albicans* Pmt1, the primary structure for these enzymes has 31 and 37% identity when compared with the fungal enzyme, suggesting similar motifs for enzyme activity but different conformations of catalytic pockets [125]. However, the effect of these compounds on human cells and in in vivo conditions remains to be addressed.

MOL3, a small molecule designed by computational docking, showed the ability to inhibit *Paracoccidioides lutzii* Kre2 and *C. albicans* Mnt1, both proteins that are involved in *O*-linked glycan synthesis [126]. With the exception of *C. glabrata*, this compound showed the ability to inhibit the growth of other *Candida* species [126]. When tested on *C. parapsilosis*, the minimal fungicidal concentration of MOL3 varied from 16 to 128 mg/L, depending on the analyzed strain [126]. When cells were incubated with 32 mg/L MOL3, they showed defects in the cell wall structure, absence of pseudohyphae when dimorphism was stimulated, and loss of cell metabolism [126]. In one cell line, MOL3 showed 30% cytotoxicity at 256 mg/L, and this was reduced when lower concentrations of the compound were incubated with the cell line, suggesting low cytotoxicity, and in vivo assays with 0.05 g/kg MOL3 applied to mice did not show any metabolic change suggestive of cytotoxicity [126]. Finally, MOL3 showed the ability to reduce the *C. parapsilosis* load in a murine model of systemic infection [126]. Collectively, these data make this small molecule a promising therapeutic candidate for the treatment of fungal infections.

Wortmannin has been isolated from *Penicillium radicum* and has shown antifungal activity against a vast range of medically relevant fungal species [127]. Despite being a phosphoinositide 3-kinase (PI3K) inhibitor, molecular docking analyses have provided confident data that suggest that this molecule may have other molecular targets, including the Kre2/Mnt1 Golgi α-1,2-mannosyltransferase [127], an enzyme relevant for *O*-linked glycan elongation. However, confirmation of this bioinformatically generated prediction is required to unveil the potential of this compound as an inhibitor of a glycosylation process.

Pradimicins A, B, C, D, and E are polyketides produced by the actinomycete *Actinomadura hibisca*, while benanomicins are produced by *Actinoallomurus spadix* [128,129]. Both types of nonribosomal peptides have shown antifungal activity in a mechanism that involves recognition of cell wall mannose, in a calcium-dependent mechanism that leads to apoptosis-like fungal cell death [130,131,132]. Interestingly, pradimicins have shown both in vitro and in vivo abilities to kill *C. albicans*, *A. fumigatus*, and *C. neoformans* [133]. Pradimicin A showed efficacy against experimental pulmonary candidiasis and aspergillosis, vaginal candidiasis, and superficial infections caused by *Trichophyton mentagrophytes* [133]. This compound was not toxic for mammalian cells at concentrations up to 500 µg/mL, which surpasses the LD_50_ of 400 mg/kg [133]. Benanomicin A showed in vivo antifungal activity against *Pneumocystis carinii* pneumonia when administered at 10 mg/kg [134].

Helja, a sunflower mannose-binding lectin belonging to the jacalin family, binds to *C. albicans* cell wall mannoproteins and, at a 200 μg/mL concentration, inhibits *C. albicans*, *C. parapsilosis*, and *C. tropicalis* growth [135]. The antifungal mechanism involves the disruption of the cell wall integrity, the inhibition of dimorphism, adhesion and biofilm formation, and the production of radical oxygen species [135,136].

Fosmanogepix (APX001), an N-phosphonooxymethyl prodrug of APX001A, targets Gwt1, an enzyme involved in the early steps of GPI anchor synthesis [112,137]. As a consequence, cell wall proteins do not reach the final destination within the cell wall, affecting both the cell wall integrity and fungal growth [92,137]. Thus far, this compound has shown antifungal activity against *C. albicans*, *C. glabrata*, *C. tropicalis*, *C. parapsilosis*, *Candida krusei*, *Candida dubliniensis*, *Candida lusitaniae*, *C. guilliermondii*, *Malassezia furfur*, *C. neoformans*, and *A. fumigatus*, with MICs ranging <0.008–0.03 µg/mL and 0.03–0.13 µg/mL for *Candida* spp. and *A. fumigatus*, respectively [93]. Moreover, fosmanogepix did not affect the activity of the Gwt1 human ortholog, and thus inositol acylation of GPI occurred in its presence, suggesting selectivity against the fungal enzyme [92]. Since fosmanogepix prevents experimental fungal infections and has been successful in clinical phase 1 studies, it is now in phase 2 clinical trials for invasive candidiasis and aspergillosis [138,139].

In addition to all these examples, it is relevant to mention that disruption of key genes involved in the protein glycosylation pathways led to changes in the cell wall that make cells more susceptible to the action of antifungal drugs, i.e., disruption of these post-translational modifications leads to hypersensitivity to antifungal drugs. Examples of these are disruption of *A. fumigatus mnn9, mnt1*, and *pmt4* [77,87,110], *C. glabrata MNN2* [140], *C. albicans DPM1, DPM2, DPM3, ALG13, RER2, MNN10*, *PMT1*, *PMT2,* and *PMT4* [21,23,78,84], and *S. cerevisiae DPM1* and *SEC59* [141].

Despite all these examples of promising new targets for antifungal drugs, these observations have to be taken with caution, as these types of statements may not be generalized to all the players in the protein glycosylation pathways and all the fungal species, *S. cerevisiae* null mutants in the *PMT* gene family, particularly the *pmt2*Δ null mutant, showed increased tolerance to PAF26, a synthetic cationic antifungal hexapeptide antifungal [142]. Moreover, Mnn1, Mnn4, and Mnn5, Golgi-resident α-mannosyltransferases involved in the synthesis of both *N*-linked and *O*-linked glycans, are required for *S. cerevisiae* tolerance to PAF26 [142]. These observations are likely to occur because the defensins and PAF26 interact with glycosylated protein targets that need post-translational modifications to bind the antifungal compounds. For the case of the antimicrobial peptide P-113Tri, derived from human histatin 5, it requires the presence of Och1 activity to display anti-*C. albicans* activity [7]. This is because most of the *C. albicans* cell wall phosphomannan is attached to the *N*-linked mannan outer chain, and Och1 catalyzes the first step in its elaboration [28,31]. Loss of the *N*-linked outer chain implies loss of most of the phosphomannan, which confers the net negative charge of the cell surface, required for the binding and internalization of P-113Tri [7]. Disruption of *C. albicans GPI7*, a mannose-ethanolamine phosphotransferase that participates in GPI anchor synthesis, led to increments in the cell wall chitin, and as a consequence, cells showed increased resistance to caspofungin [143].

## 8. Protein Glycosylation as a Target for the Development of Antifungal Vaccines

Currently, no vaccine has been approved to treat fungal infections, and most of the experimental designs to generate immunological protection are based on epitopes that are part of key proteins for fungal pathogenesis or structural polysaccharides, such as β-1,3-glucans or chitosan [144,145]. However, some approaches that exploit the particular structure and composition of cell wall *N*-linked and *O*-linked glycans have been reported.

The β-1,2-mannotriose found as part of the *C. albicans* cell wall phosphomannan [24] was chemically synthesized and conjugated to the N-terminal end of the adhesin Als1 [146]. When injected into mice, this conjugate stimulated the production of a specific IgG against both the trisaccharide and the peptide, in an adjuvant-free event [146]. Since these antibodies recognized the trisaccharide present in the *C. albicans*, *C. tropicalis*, *C. lusitaniae*, and *C. glabrata* wall surface, it is thought that the trisaccharide alone or conjugated to Als1 could be a candidate for the development of a vaccine against these pathogens [146]. A similar approach has also been reported, conjugating this trisaccharide to fructose-bisphosphate aldolase and to β-1,3-linked hexaglucan, in order to provide epitopes that stimulate both B and T lymphocytes, thus improving the immunological properties of the conjugate [147]. The heptasaccharide β-D-Man-(1→2)-α-D-Man-(1→3)-α-DMan-(1→2)-α-D-Man-(1→3)-α-D-Man-(1→2) α-D-Man-(1→2)-α-D-Man, which belongs to the *C. albicans* antigenic factor 9, part of the *N*-linked glycan outer chain [24], was conjugated to bovine serum albumin and subcutaneously inoculated in mice [148]. After two boosts, this glycoconjugate elevated the antifungal activity of polymorphonuclear leukocytes, increased the phagocytic activity and respiratory burst of granulocytes, and elevated the subpopulations of CD3(+) T, CD4(+)CD25(+) T, and CD4(+)/CD8(+) T lymphocytes, promoting an increment in the levels of CD8(+)CD25(+) cells and a Th1-based, Th2-based, and Th17-based immune response [148]. A similar antigenic preparation (α-1, 6-linked *N*-linked glycan outer chain backbone highly branched with α-1,2-, α-1,3-, and β-1,2-linked mannooligomers) derived from *C. albicans* showed conferred protection to mice against a lethal dose of *A. fumigatus* [149]. This protection was achieved with a dose of 12 mg of mannan or with 0.3 mg when conjugated to bovine serum albumin [149]. Despite the fact that these different structures are capable of conferring resistance to antifungal infections, reverse engineering approaches have revealed that β-1,2-mannobiose conjugated to a protein is the minimal epitope to induce protective antibodies against a fungal infection [150]. Indeed, immunized rabbits with this glycoconjugate showed protection against *C. albicans* [150].

The *C. albicans* cell wall mannan extracted and encapsulated in liposomes or conjugated to a protein induced the production of protective antibodies in a murine model of systemic candidiasis, preventing animal death [151]. The mannan–protein conjugate conferred 100% protection to animals without a functional complement system, while encapsulated mannan in liposomes was capable of protecting only 60% of the animal population that received this formulation [151]. Since the administration of the mannan–protein conjugate showed superior protective levels in complement-deficient elements compared to liposomes, this formulation results in being more attractive for further development of an anti-*Candida* vaccine.

Recently, passive immunization with C3.1, an anti-β-1,2-mannotriose monoclonal antibody, showed protection in a murine model of systemic candidiasis by *Candida auris* [152], suggesting this could be an effective alternative in cases of resistance to antifungal drugs or for the treatment of immunocompromised patients.

## 9. Conclusions

In pathogenic fungi, there is a clear correlation between proper protein glycosylation and virulence. Many processes that are essential to establish diseases, such as adhesion, proliferation, dimorphism, evasion of the host immune response, and biofilm formation, depend on the correct synthesis of *N*-linked and *O*-linked glycans and GPI anchors, as demonstrated by the deletion of key genes participating in these biosynthesis pathways. In addition, glycosylation plays an important role in cell wall synthesis, a process that influences the cell shape, growth rate, and wall plasticity and contributes to general cell fitness. Therefore, knowledge about the genes and proteins involved in these metabolic routes is of particular relevance in medically relevant fungal species, as it can lead to developing new therapeutic approaches to treat mycoses. As exemplified here, protein glycosylation could be considered as a promising target not only for antifungal drugs but also for the development of antifungal vaccines. One imperative challenge in the short and middle terms is to analyze these pathways in other relevant fungal species. Despite the fact that our knowledge about glycan structures and enzymes in charge of their elaboration can be considered vast, it is narrow when listing the fungal species where these aspects have been studied. It is now clear that glycan structures cannot be extrapolated from one species to another, and a similar observation applies to the contribution of enzymes to glycan synthesis, underlining the need for more studies in this area.

## Figures and Tables

**Table 1:** Phenotype of mutant strains in genes involved in *N*-linked or *O*-linked glycosylation, glycosylphosphatidylinositol anchor synthesis, and non-conventional glycosylation processes, in medically relevant fungal species.

Gene	Mutant Phenotype	Impact on Virulence	References
*N-*Linked Glycosylation			
*CWH41*	*Candida albicans* Cellular aggregates, defects in cell wall composition. *Aspergillus fumigatus* Reduction in conidia formation, changes in the cell wall composition, polar growth, and hyphal elongation.	Attenuated virulence in a murine model. None found.	[26,61]
*ROT2*	*C. albicans* Cellular aggregates, defects in cell wall composition. *Candida glabrata* None found.	Attenuated virulence in a murine model. Decreased virulence in *Drosophila melanogaster*.	[26,62]
*MNS1*	*C. albicans* Few phenotypical changes. *C. glabrata* None found.	Virulence affected. Decreased virulence in a murine model of systemic candidiasis.	[26,62,63]
*OCH1*	*C. albicans* Defects in the cell wall, cellular aggregates, decreased growth rate, and reduced ability to stimulate cytokine production. *Candida parapsilosis* Changes in cell morphology and cell wall composition, susceptibility to cell wall perturbing agents, and reduced ability to stimulate cytokine production. *Sporothrix schenckii* Minimal changes in the phenotype and the cell wall. *A. fumigatus:* Defects in ability to sporulate, non-significant changes in phenotype. *Cryptococcus neoformans* None found.	Attenuated virulence in a murine model. Attenuated virulence in neonate and adult murine models. Attenuated virulence in a murine model and the invertebrates *Galleria mellonella* and *Tenebrio molitor*. None found. Slightly attenuated virulence.	[26,64,65,66,67,68,69,70]
*ANP1*	*C. glabrata* None found.	Decreased virulence in both murine model of systemic candidiasis and *D. melanogaster*.	[62]
*MNN14*	*C. albicans* Defects in cell wall composition, but normal ability to stimulate cytokine production.	Virulence attenuation in a murine model of disseminated candidiasis.	[71]
*MNN2*	*C. albicans* Changes in phenotype.	Attenuated virulence in *G. mellonella* and a murine model.	[72]
*MNN4*	*C. albicans* Changes in the *C. albicans*–macrophage interaction. *Candida tropicalis* Changes in the yeast–macrophage interaction. *C. glabrata* None found.	Attenuated virulence in the *G. mellonella* model but not in a murine model. Attenuated virulence in *G. mellonella* model. Attenuated virulence in a murine model of systemic candidiasis.	[30,62,73,74]
*MNN5*	*C. albicans* Hypersensitivity to wall perturbing agents, inability to undergo dimorphism.	Attenuated virulence in a murine model.	[75]
*MNN9*	*C. albicans* Changes in the cell wall, osmotic instability, defective hyphae, and cell agglutination. *A. fumigatus* Defects in the cell wall, changes in morphogenesis, sensitivity to wall perturbing agents.	None found. None found.	[76,77]
*MNN10*	*C. albicans* Abnormal organization of the cell wall. *C. glabrata* Abnormal growth in vitro	Decreased virulence. Decreased virulence in both murine model of systemic candidiasis and *D. melanogaster*	[62,78]
*MNN11*	*C. glabrata* None found.	Virulence is not affected.	[79]
*KRE2/MNT1*	*C. albicans* Cellular aggregates, susceptibility to cell wall perturbing agents, reduction in the ability to stimulate cytokine production. *C. glabrata* None found.	Attenuated virulence in a murine model. Virulence attenuation in both a murine model of candidiasis and *D. melanogaster*.	[31,62]
*KTR3*	*C. glabrata* None found.	Virulence attenuation in *D. melanogaster*.	[62]
*PMR1*	*C. albicans* Cellular aggregates, defects in the cell wall composition, hypersensitivity to wall perturbing agents. *Candida guilliermondii* Affected cell growth, morphology, cell wall, biofilm formation, and interaction with PBMC. *A. fumigatus* Defects in growth, changes in the cell wall composition, increased content of β-glucan and chitin. Hypersensitivity to cell wall inhibitors.	Attenuated virulence in a murine model. Attenuated virulence in a murine model and *G. mellonella*. None found.	[80,81,82]
*ALG6*	*C. glabrata* None found.	Virulence attenuation in *D. melanogaster*.	[62]
*ALG13*	*C. albicans* Defects in the hypha and biofilm formation, changes in the cell wall carbohydrate content. Defects in *N*-linked glycosylation.	None found.	[23]
*DPM1*	*C. albicans* Susceptibility to wall perturbing agents, altered cell wall composition and affected growth, increased chitin levels.	None found.	[21]
*DPM2*	*C. albicans* Susceptibility to wall perturbing agents and changes in the cell wall integrity.	None found.	[21]
*DPM3*	*C. albicans* Growth rate attenuation and increased chitin levels.	None found.	[21]
*SRB1*	*C. albicans* Changes in morphology, growth, sensitivity to antifungal agents and cell wall inhibitors, and glycosylation defects.	None found.	[83]
*O*-Linked Glycosylation			
*PMT1*	*C. albicans* Hypersensitivity to antifungal agents and changes in the cell wall components. *C. glabrata* None found. *A. fumigatus* Growth defects, changes in the cell wall, defects in cell morphology, and conidia formation. *C. neoformans:* None found.	Severely affected virulence in a murine model. Virulence attenuation in *D. melanogaster*. None found. Attenuated virulence in a macrophage death model and a murine model.	[62,84,85,86,87,88]
*PMT3*	*C. glabrata* None found.	Decreased virulence in a murine model of systemic candidiasis.	[62]
*PMT4*	*C. albicans* Hypersensitivity to antifungal agents and changes in the cell wall components. *C. glabrata* None found. *A. fumigatus* Growth defects, changes in the cell wall, defects in cell morphology, and conidia formation. *C. neoformans:* Morphological defects, alterations in the cell wall.	Severely affected virulence in a murine model. Decreased virulence in a murine model of systemic candidiasis. None found. Virulence affected.	[62,84,86,87,88,89]
*PMT5*	*C. albicans:* Defects in morphogenesis and susceptibility to antifungal agents.	None found.	[84]
*PMT6*	*C. albicans:* None found.	Severely affected virulence in a murine model.	[85,86]
*MNT1*	*C. albicans:* Changes in cell morphology, inability to adhere to human buccal epithelial cells, hypersensitivity to wall perturbing agents. *A. fumigatus:* Reduction in the cell wall thickness, decreased cell growth.	Attenuated virulence in a murine model. Attenuated virulence.	[34,90]
*MNT2*	*C. albicans:* Few defects in phenotype.	Attenuated virulence.	[34]
Glycosylphosphatidylinositol Anchors			
*GWT1*	*Candida* spp., *Aspergillus* spp., *Fusarium* spp., and *Scedosporium* spp Changes in the cell wall integrity, structure, growth, and dimorphism. *C. albicans:* Growth and morphological changes, reduced adherence, growth, and dimorphism.	Virulence affected. None found.	[6,91,92,93,94]
*GPI7*	*C. albicans* Effects on chlamydospore formation, altered levels of plasma membrane GPI proteins, abnormal polarity, and growth.	None found.	[95]
*GPI15*	*C. albicans* Presents hypofilamentous, ergosterol defective, and azole sensitive.	Virulence affected, less virulence in vivo.	[96]
*GPI3*	*A. fumigatus* Growth rate significantly slower, sensitivity to SDS, increased levels of the chitinase ChiB.	Reduced virulence in a murine model.	[97]
Non-Conventional Glycosylation Processes			
*glfB*	*A. fumigatus* Poor growth.	Attenuated virulence in a murine model.	[45,98]
*umg1*	*A. fumigatus* Thinner cell wall, susceptibility to antifungal agents, and hyper-adherent cells.	None found.	[99]
*ktr4* and *ktr7*	*A. fumigatus* Severe phenotype defects, showing hyperbranched mycelium, loss of polarity during conidia germination, and hyphal elongation.	Virulence affected.	[46]
*KTR3*	*C. neoformans* Sensitivity to SDS and NaCl.	Attenuated virulence in a murine model.	[54]
*RmlD*	*S. schenckii* Few phenotypical changes, alterations in the cell wall composition and organization.	Attenuated virulence in the *G. mellonella* model.	[57]

## Data Availability

Not applicable.
